# Drug Discovery Based on Oxygen and Nitrogen (Non-)Heterocyclic Compounds Developed @LAQV–REQUI*M*TE/Aveiro

**DOI:** 10.3390/ph16121668

**Published:** 2023-11-30

**Authors:** Joana L. C. Sousa, Hélio M. T. Albuquerque, Artur M. S. Silva

**Affiliations:** LAQV-REQUI*M*TE, Department of Chemistry, University of Aveiro, 3810-193 Aveiro, Portugal; joanasousa@ua.pt (J.L.C.S.); helio.albuquerque@ua.pt (H.M.T.A.)

**Keywords:** medicinal chemistry, drug discovery, heterocyclic compounds, polyphenols, antioxidant, anti-inflammatory, antidiabetic, anticancer, anti-Alzheimer, bioimaging

## Abstract

Artur Silva’s research group has a long history in the field of medicinal chemistry. The development of new synthetic methods for oxygen (mostly polyphenols, e.g., 2- and 3-styrylchromones, xanthones, flavones) and nitrogen (e.g., pyrazoles, triazoles, acridones, 4-quinolones) heterocyclic compounds in order to be assessed as antioxidant, anti-inflammatory, antidiabetic, and anticancer agents has been the main core work of our research interests. Additionally, the synthesis of steroid-type compounds as anti-Alzheimer drugs as well as of several chromophores as important dyes for cellular imaging broadened our research scope. In this review article, we intend to provide an enlightened appraisal of all the bioactive compounds and their biological properties that were synthesized and studied by our research group in the last two decades.

## 1. Introduction

**Figure 1:**
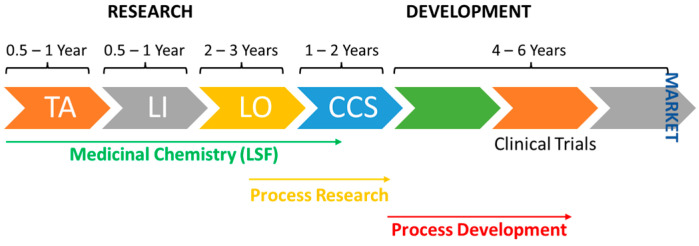
Schematic depiction of drug discovery process. TA: target assessment; LI: lead identification; LO: lead optimization; CCS: clinical candidate selection; LSF: late-stage functionalization.

Medicinal chemistry is a chemistry-based discipline, which has the primary objective of designing and discovering new compounds suitable for use as drugs [1]. However, this simplistic definition ignores the larger historical context of medicinal chemistry and its ongoing evolution. In 1998, IUPAC proposed a more holistic definition for medicinal chemistry—“Medicinal Chemistry is a chemistry-based discipline, also involving aspects of biological, medical, and pharmaceutical sciences. It is concerned with the invention, discovery, design, identification and preparation of biologically active compounds, the study of their metabolism, the interpretation of their mode of action at the molecular level and the construction of structure-activity relationships (SARs)”. Medicinal chemistry is a highly interdisciplinary science combining several fields such as organic chemistry, biochemistry, computational chemistry, pharmacology, molecular biology, statistics, and physical chemistry, among others. Medicinal chemists play a crucial role in the drug discovery process, especially in its early stages (Figure 1). This requires a thorough understanding of modern organic chemistry, essential to prepare new chemical entities [2]. The role of organic chemistry is not limited to the early stages of drug development. In recent years, with the advent of C-H activation, medicinal chemists were driven to use late-stage functionalization (LSF) strategies, which enable a rapid exploration of SARs, the generation of oxidized metabolites, the blocking of metabolic hot spots, and the preparation of biological probes (Figure 1). LSF strategies created the possibility of exploring the chemical space more effectively than the conventional synthetic approaches [3,4].

Heterocyclic compounds are defined as cyclic structures with at least one heteroatom. Among all the possible heteroatoms, nitrogen, oxygen, and sulfur are most prevalent ones in heterocyclic compounds. In the medicinal chemistry field, the role of heterocycles is quite indisputable as they are often present not only as fragments in a number of active pharmaceutical ingredients (APIs) but also as excipients. The importance of heterocycles in medicinal chemistry is often related to their capacity to modify physicochemical properties (solubility, lipophilicity, polarity, and hydrogen bonding capacity of biologically active agents), essential for the optimization of the absorption, distribution, metabolism, and excretion–toxicity (ADMET) properties of drugs and drug candidates.

It is estimated that up to 85% of biologically active compounds have in their structure a heterocyclic fragment. In terms of approved drugs, 84% have at least one nitrogen atom in their scaffolds, and about 60% have some sort of nitrogen heterocycle such as piperidine, pyridine, pyrrolidine, thiazole, imidazole, indole, and tetrazole [5,6]. Oxygen heterocycles are the second most prevalent type of heterocycle in approved drugs, with pyranoses, furanoses, macrolactones, morpholines, and dioxolanes being placed in the top five examples [7].

**Figure 2:**
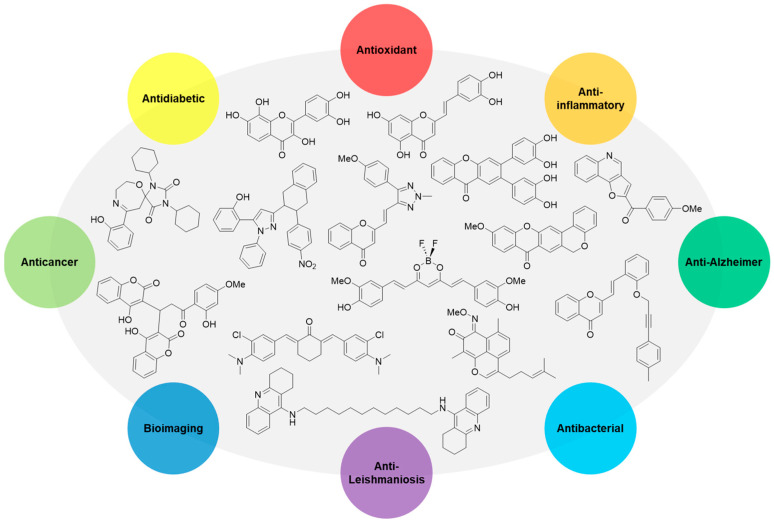
Schematic overview of some developed bioactive compounds as antioxidant, anti-inflammatory, antidiabetic, anticancer, anti-Alzheimer, bioimaging, anti-Leishmaniosis and antibacterial agents.

In this personal account, around twenty years of development of bioactive compounds will be reviewed, focusing not only on traditional biological activities like antioxidant, anti-inflammatory, antidiabetic, and anticancer properties, but also more recent interests such as Alzheimer’s disease-targeting compounds and bioimaging tools (Figure 2).

## 2. Antioxidant Activity

### 2.1. ROS/RNS Scavenging Activity

The main contributing factor to the oxidative stress-related pathologies, such as inflammation, atherosclerosis, cancer, and aging, is the overproduction of reactive oxygen species (ROS) and reactive nitrogen species (RNS). The maintenance of the ROS/RNS balance is carried out by endogenous enzymatic antioxidant defenses such as superoxide dismutase, glutathione peroxidase, thioredoxin reductase, and catalase, and by non-enzymatic compounds such as glutathione, uric acid, and coenzyme Q. If the internal production of antioxidants is not enough to neutralize all the ROS/RNS produced, a series of exogenous non-enzymatic antioxidants can be provided from the human diet that includes carotenoids, phenolic compounds, and flavonoids, among others.

**Figure 3:**
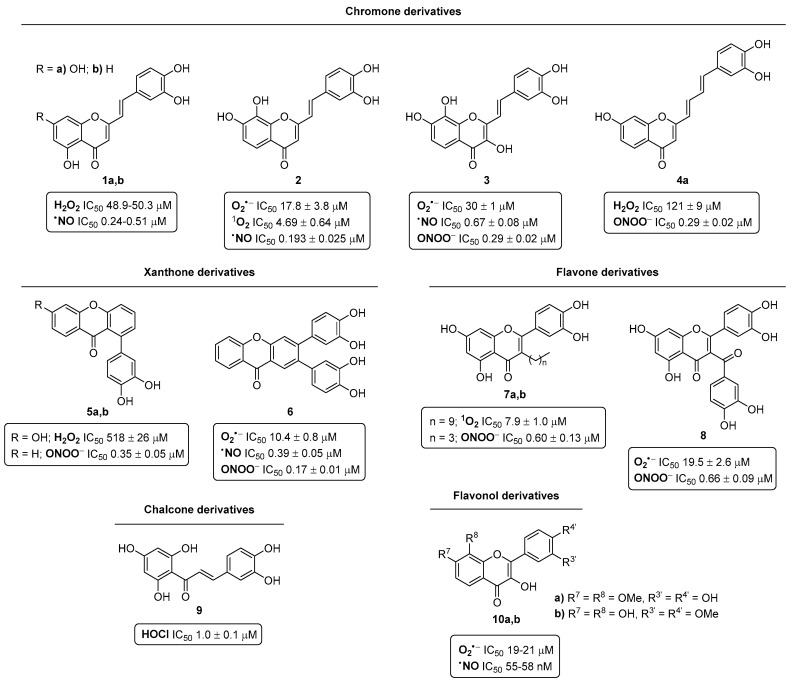
Most efficient scavengers of ROS and RNS based on polyhydroxylated 2-[styryl or (4-arylbutadienyl)]chromones **1**–**4**, xanthones **5**,**6**, flavones **7**,**8**, chalcones **9**, and flavonols **10**.

Based on the demand for new antioxidant agents, the design and synthesis of more effective scavengers of ROS and RNS was the core of our research for many years [8,9,10,11,12,13]. In this context, we synthesized novel polyhydroxylated 2-[styryl or (4-arylbutadienyl)]chromones **1**–**4**, xanthones **5**,**6**, flavones **7**,**8**, chalcones **9**, and flavonols **10** (Figure 3), which were assessed for their in vitro scavenging capability against the most physiologically relevant ROS (superoxide radical (O_2_^•−^), hydrogen peroxide (H_2_O_2_), hypochlorous acid (HOCl), singlet oxygen (^1^O_2_), and peroxyl radical (ROO^•^)) and RNS (nitric oxide (^•^NO) and peroxynitrite anion (ONOO^−^)).

In the Figure 3, the active derivatives of each family to scavenge the referred to ROS and RNS are summarized in order to show which type of structures are more suitable for scavenging each reactive species. Thus, among the active compounds **1**,**4**,**5** to scavenge H_2_O_2_, the 2-styrylchromone (2-SC) derivatives **1a**,**b**, presenting a catechol moiety at the B-ring in conjugation with the Cα=Cβ double bond of the styryl moiety, and free OH groups at C-5 and C-7, emerged as lead compounds with IC_50_ values of 48.9–50.3 µM (in comparison with ascorbic acid used as positive control, IC_50_ = 625.5 ± 49.6 µM) [13]. On the other hand, the 2-SC derivative **2**, possessing two catechol units at the A- and B-rings, was more effective for scavenging ^1^O_2_ (IC_50_ = 4.69 ± 0.64 µM) than ascorbic acid, used as a positive control (IC_50_ = 10.2 ± 1.5 µM) [12], and the flavones **7**. In addition, the xanthone **6**, bearing two catechol units at C-2 and C-3, is a potent scavenger of O_2_^•−^ (IC_50_ = 10.4 ± 0.8 µM) vs. tiron, used as a positive control (IC_50_ = 273 ± 32 µM), and compounds **2**,**3**,**8**,**10**. The xanthone **6** was also an effective scavenger of ONOO^−^, showing an IC_50_ value of 0.17 ± 0.01 µM vs. ebselen, used as a positive control (IC_50_ = 0.50 ± 0.03 µM) [11], and compounds **3**–**5**,**7**,**8**. Regarding the scavenging activity against HOCl, the chalcones **9** were the most active scavengers, specifically, the derivative bearing a catechol moiety at the B-ring and free OH groups at C-2′, C-4′, and C-6′ was the most potent compound (IC_50_ = 1.0 ± 0.1 µM) in comparison with ascorbic acid, used as a positive control (IC_50_ = 11 ± 1 µM) [8]. Finally, to scavenge ^•^NO, the flavonols **10a**,**b** were definitely the most potent compounds, as they are active in the nanomolar range (IC_50_ = 55–58 nM) compared to quercetin, used as a positive control (IC_50_ = 1.3 ± 0.1 µM) [9], and to compounds **1**–**3**,**6**.

### 2.2. Free Radicals Scavenging Activity

**Figure 4:**
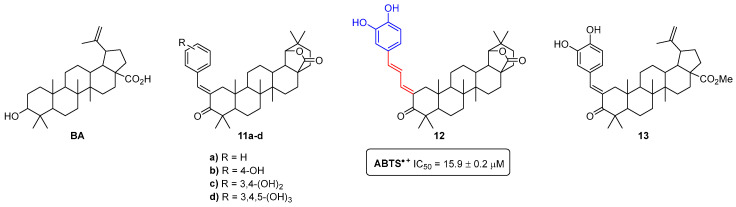
Chemical structures of the betulinic acid (BA) and the 19,28-epoxyoleanane-3,28-dione and methyl betulonate-derived polyhydroxylated compounds **11**–**13**.

Using betulinic acid (BA), which is a lupane-type pentacyclic triterpenoid commonly isolated from the bark of birch trees, as a raw material to produce amphiphilic antioxidants, we synthesized the polyhydroxylated 19,28-epoxyoleanane-3,28-dione-type **11a**–**d** and **12**, and methyl betulonate-type **13** compounds (Figure 4) [14]. We found that the derivative **12**, bearing a catechol moiety and an extended π-conjugated carbonyl system, emerged as a lead compound, since it was revealed to be the most efficient scavenger of the 2,2′-azino-bis(3-ethylbenzothiazoline-6-sulfonic acid) radical cation (ABTS^•+^) (IC_50_ = 15.9 ± 0.2 µM). In fact, it was more active than α-tocopherol (IC_50_ = 19.2 ± 0.1 µM), used as a positive control and the pristine BA (no activity was found up to the highest tested concentration—800 µM).

**Figure 5:**
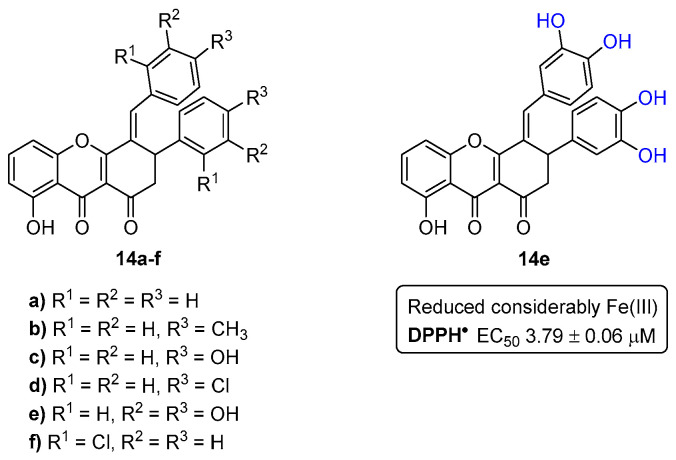
Chemical structures of the xanthene-1,9(2*H*)-diones **14a**–**f** and the most active derivative **14e** in reducing the ferric ion and scavenging DPPH^•^.

The antioxidant activity of xanthenediones **14a**–**f** (Figure 5) was evaluated by their ability to scavenge the 2,2-diphenyl-1-picrylhydrazyl free radical (DPPH^•^) and to reduce Fe(III) [15]. The compounds were tested at different concentrations and, naturally, the reducing ability increased with concentration. Among the tested compounds, the derivative **14e** reduced considerably the ferric ion, being more efficient than 2,6-bis(1,1-dimethylethyl)-4-methylphenol (BHT) and similar to quercetin, used as positive controls. This result confirmed that a catechol moiety in a molecule is important for the reducing power activity. In addition, this derivative was also the most potent scavenger of DPPH^•^, presenting an EC_50_ value of 3.79 ± 0.06 µM. Furthermore, these compounds were also evaluated for their anti-acetylcholinesterase activity, which will be discussed later in Section 6.

**Figure 6:**
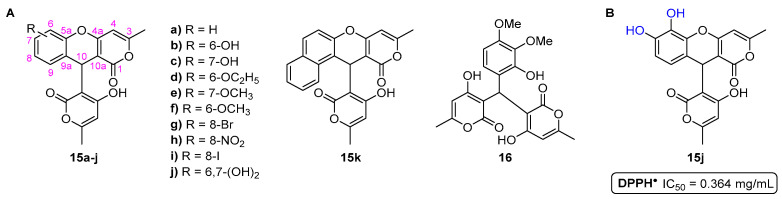
Chemical structures of the 10-(4-hydroxy-6-methyl-2-oxo-2*H*-pyran-3-yl)-3-methyl-1*H*,10*H*-pyrano[4,3-*b*]chromen-1-ones **15a**–**k** and 3,3′-[(2-hydroxy-3,4-dimethoxyphenyl)methylene]bis(4-hydroxy-6-methyl-2*H*-pyran-2-one) (**16**) (**A**) and the most active compound **15j** in the scavenging of DPPH^•^ (**B**).

The antioxidant activity of the synthesized 10-(4-hydroxy-6-methyl-2-oxo-2*H*-pyran-3-yl)-3-methyl-1*H*,10*H*-pyrano [4,3-*b*]chromen-1-ones **15a**–**k** and 3,3′-[(2-hydroxy-3,4-dimethoxyphenyl)methylene]bis(4-hydroxy-6-methyl-2*H*-pyran-2-one) **16** (Figure 6) was evaluated using the DPPH^•^ scavenging assay [16]. Compound **15j**, bearing a catechol moiety at the A-ring, was the most active compound, presenting an IC_50_ value of 0.364 mg/mL.

### 2.3. Enzymatic Inhibition Activity

Xanthine oxidase (XO) is a highly versatile enzyme with a broad range of activities in reducing substrates. As a consequence, XO is considered an important biological source of ROS, inducing oxidative stress, and being involved in many pathological processes. Therefore, the development of XO inhibitors is expected to be therapeutically useful for the treatment of the aforementioned pathological states.

In this context, a series of 2-SCs developed in our group were evaluated as potential XO inhibitors [17]. From a library of ten 2-SCs, the results showed a concentration-dependent and non-competitive inhibition effect, with derivative **1a** (Figure 3) being the most potent compound (IC_50_ = 0.55 ± 0.03 μM), 10-fold more effective than the positive control, allopurinol (IC_50_ = 5.43 ± 0.80 μM).

## 3. Anti-Inflammatory Activity

### 3.1. Flavones

**Figure 7:**
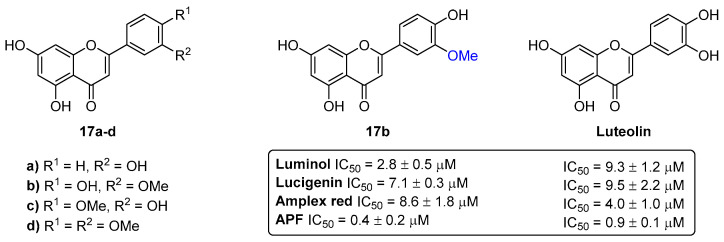
Chemical structures of the methylated flavones **17a**–**d** and the most effective derivative **17b** in inhibiting PMA-induced neutrophils’ oxidative burst.

Methylated flavones **17a**–**d** (Figure 7) have revealed the ability to modulate the neutrophils’ oxidative burst [18]. Thus, the study of the modulatory effect of **17a**–**d** on the stimulation of neutrophils by the phorbol-12-myristate-13-acetate (PMA) involved fluorescent [using amplex red and aminophenylfluorescein (APF) as probes] and chemiluminescent (using luminol and lucigenin as probes) techniques. The flavone **17b** was the most potent derivative (IC_50_ = 0.4–8.6 µM) in inhibiting PMA-induced neutrophils’ oxidative burst assessed by both chemiluminescent and fluorescent techniques (Figure 7). These results suggest that the 3′-methoxy group in the B-ring is an important chemical feature that contributed to its higher activity in comparison to luteolin (IC_50_ = 0.9–9.5 µM).

These flavone derivatives were also evaluated as cyclooxygenase (COX) and lipoxygenase (LOX) inhibitors [19,20]. However, these compounds did not show any promising results when compared to other flavonoid-type compounds.

**Figure 8:**
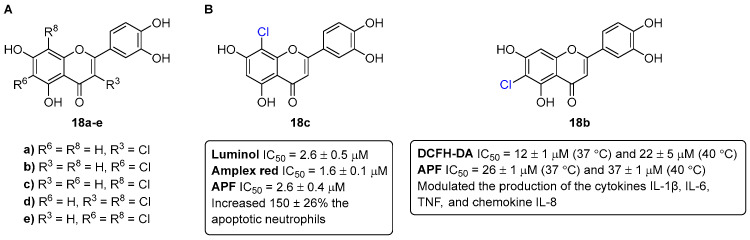
Chemical structures of the chlorinated flavones **18a**–**e** (**A**) and the lead chlorinated flavones as anti-inflammatory agents (**B**). DCFH-DA = 2′,7′-Dichlorodihydrofluorescein diacetate.

The novel chlorinated flavones **18a**–**e** (Figure 8) were investigated for their anti-inflammatory properties in comparison with the parent non-chlorinated flavonoids [21,22].

Firstly, their effect in neutrophils’ oxidative burst and lifespan was studied [21]. The obtained results demonstrate that chlorinated flavonoids were more efficient than their parent compounds in modulating neutrophils’ oxidative burst in PMA-activated neutrophils. Some of the tested flavonoids drive neutrophil apoptosis in a caspase 3-dependent fashion. The present data showed that 8-chloro-3′,4′,5,7-tetrahydroxyflavone (**18c**) constitutes an alternative anti-inflammatory therapy, due to the proven ability to suppress mechanisms engaged at the onset and progression of inflammation (Figure 8).

Then, the anti-inflammatory potential of these chlorinated flavones **18a**–**e** (Figure 8) were also tested in the activity of COX-1 and COX-2, and in the production of cytokines (interleukins (IL-6 and IL-1β) tumor necrosis factor (TNF)), and the chemokine IL-8, as well as in the production of reactive species, using human whole blood as a representative in vitro model [22]. None of the chlorinated flavones **18a**–**e** were able to inhibit COX at the highest tested concentration (100 μM). However, 6-chloro-3′,4′,5,7-tetrahydroxyflavone (**18b**) was able to reduce the production of reactive species, even in hyperthermic conditions, and also modulated the production of the cytokines IL-1β, IL-6, TNF, and the chemokine IL-8b (Figure 8).

### 3.2. Xanthones

**Figure 9:**
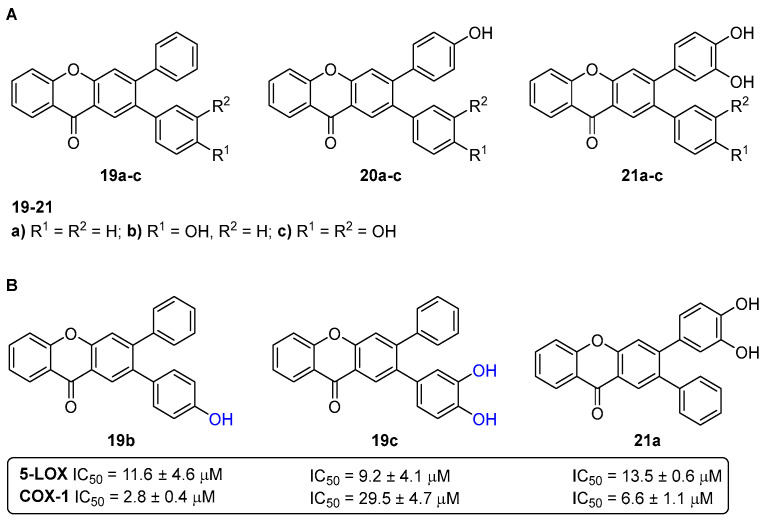
Chemical structures of the hydroxylated 2,3-diarylxanthones **19**–**21** (**A**) and the emerged dual-acting anti-inflammatory agents **19b**,**c** and **21a** (**B**).

With the demand for new dual-acting anti-inflammatory agents, a range of 2,3-diarylxanthones **19**–**21** (Figure 9) were tested through their ability to interact in arachidonic acid metabolism [23]. The in vitro anti-inflammatory activity was evaluated through the inhibition of 5-LOX-catalyzed leukotriene B_4_ (LTB_4_) formation in human neutrophils and the inhibition of COX-1- and COX-2-catalyzed prostaglandin E_2_ (PGE_2_) formation in human whole blood.

The xanthone **19c** with a 2-catechol group was the most active one (IC_50_~9 μM) in preventing LTB_4_ production in human neutrophils. The more effective arylxanthones in preventing COX-1-catalyzed PGE_2_ production presented IC_50_ values from 1 to 7 μM, exhibiting a structural feature with at least one non-substituted aryl group. All the studied arylxanthones were ineffective at preventing the formation of PGE_2_ catalyzed by COX-2, up to the maximum concentration of 100 μM. The ability of the tested 2,3-diarylxanthones to interact with both 5-LOX and COX-1 pathways constitutes an important step in the research of novel dual-acting anti-inflammatory drugs (Figure 9).

### 3.3. 2-Styrylchromones

The anti-inflammatory potential of 2-SCs was evaluated by studying their COX-1 and COX-2 inhibitory capacity as well as their effects on the LTB_4_ production using stimulated human polymorphonuclear leukocytes (PMNL) [24].

Some of the tested 2-SCs were able to inhibit both COX-1 activity and LTB_4_ production, which makes them dual inhibitors of the COX and 5-LOX pathways. The most effective compound in this study was derivative **1a** (Figure 3), which has structural moieties with proven antioxidant activity (free OH groups at C-5 and C-7 and a 3′,4′-catechol-substituted B-ring in conjugation with the Cα=Cβ double bond of the 2-styryl moiety).

This type of compound may exhibit anti-inflammatory activity with a wider spectrum than that of classical non-steroidal anti-inflammatory drugs (NSAIDs) by inhibiting 5-LOX product-mediated inflammatory reactions, towards which NSAIDs are ineffective.

## 4. Antidiabetic Activity

Diabetes mellitus (DM) is one of the most significant public health concerns worldwide. According to the International Diabetes Federation, 10.5% of the adult population (20–79 years) has diabetes according to 2021 data, and will probably reach 783 million (1 in 8 adults) in 2045, an increase of 46% [25]. DM is a multifactorial metabolic disorder, characterized by chronic hyperglycemia and can be primarily classified as type 1 (T1DM, insulin-dependent DM) and type 2 (T2DM, non-insulin-dependent DM) [26]. T2DM is the most common form of DM, accounting for more than 90% of all diabetic patients, and results from the interaction between behavioral, environmental, and genetic risk factors [27,28].

Novel therapeutic druggable targets for the management of T2DM have been emerging, namely carbohydrate-hydrolyzing enzymes α-amylase and α-glucosidase, fructose-1,6-bisphosphatase (FBPase), protein tyrosine phosphatase 1B (PTP-1B), dipeptidyl peptidase-4 (DPP-4) and glycogen phosphorylase (GP). The discovery of new inhibitors of these targets represents an alternative for the currently used antidiabetic agents. In the next subsections, we will show the potential of synthetic flavonoid, 2-(styryl or 4-arylbutadienyl)chromone, xanthone, and pyrazole derivatives as antidiabetic agents through inhibiting some of the referred to targets.

### 4.1. Flavonoids

#### 4.1.1. Flavonols

**Figure 10:**
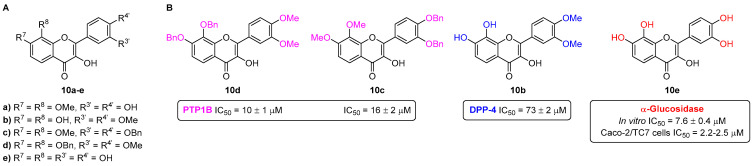
Chemical structures of flavonols **10a**–**e** (**A**) and the most active flavonols for the in vitro inhibition of PTP1B, DPP-4, and α-glucosidase (**B**).

The flavonols **10a**–**e** were designed to possess catechol moieties at the A-ring and/or B-ring (Figure 10) [9], allowing the assessment of SAR studies involving these compounds as antidiabetic agents, namely, as DPP-4, PTP1B, FBPase, and α-glucosidase inhibitors.

The flavonols **10a**–**e** were found to be effective PTP1B inhibitors for the treatment of T2DM [26]. The most active derivatives were **10d** (IC_50_ = 10 ± 1 µM), followed by **10c** (IC_50_ = 16 ± 2 µM), showing that the presence of both -OBn and -OMe groups in the flavonol structure significantly increases their in vitro PTP1B inhibition (Figure 10). In comparison with the flavonol **10e**, which possesses five -OH groups in their place, its ability to inhibit PTP1B was much lower (%inhibition = 36 ± 3% at the highest tested concentration of 200 μM).

The flavonols **10a**–**e** were studied concerning their ability to inhibit DPP-4 enzyme [27]. This study was based on in vitro fluorometric and colorimetric methods using the human isolated enzyme. The fluorometric method was revealed to be more sensitive and was applied in the evaluation of DPP-4 activity in an ex vivo assay, using human blood and plasma. The only effective flavonol based on the fluorometric method was **10b** (IC_50_ = 73 ± 2 µM) (Figure 10), displaying no inhibitory activity in the ex vivo method, probably due to its high affinity for plasma proteins, notably, human serum albumin.

The same flavonols **10a**–**e** were also tested as FBPase inhibitors [28]. However, none of these derivatives performed as effective FBPase inhibitors, presenting a %inhibition <20% at the highest tested concentration of 200 µM.

The flavonols **10a**–**e** were screened for their in vitro inhibitory activity of α-glucosidase [29]. In this case, flavonol **10e** was the most active derivative (Figure 10), presenting an IC_50_ (7.6 ± 0.4 µM) much lower than the one found for the most widely prescribed α-glucosidase inhibitor, acarbose (IC_50_ = 607 ± 56 µM). Due to this promising result, the flavonol **10e** was further studied for its inhibitory activity against human sucrase-isomaltase, the α-glucosidase found in Caco-2/TC7 cells [30], being the most active compound among the tested flavonoids (Figure 10), presenting an IC_50_ value of 2.2 ± 0.2 μM and 2.5 ± 0.2 μM when using sucrose or maltose as substrates, respectively.

#### 4.1.2. Chlorinated Flavones

**Figure 11:**
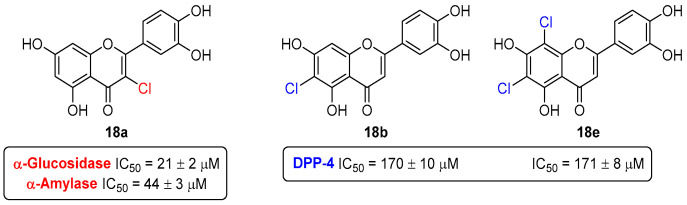
The most active chlorinated flavones as α-glucosidase, α-amylase, and DPP-4 inhibitors.

The chlorinated flavones **18a**–**e** (Figure 8) were explored for their inhibitory effect against α-glucosidase for the first time [29]. All of them were active, except for **18b**, presenting IC_50_ values of 21 ± 2 to 55 ± 2 µM, being more potent than acarbose, used as a positive control (IC_50_ 607 ± 56 µM). Among this group of compounds, the 3-chlorinated derivative **18a** was the most active compound (Figure 11), presenting an IC_50_ (21 ± 2 µM) similar to the one obtained for quercetin (15 ± 3 µM), showing that the presence of a 3-OH group or a 3-Cl atom at the C-ring is almost indifferent for the inhibitory effect.

This group of chlorinated flavones **18a**–**e** was also addressed for their inhibitory pancreatic α-amylase activity [31]. The 3-chlorinated derivative **18a** was once again the most active compound (Figure 11), presenting an IC_50_ value of 44 ± 3 µM. In this case, the presence of a 3-Cl atom at the C-ring seems to be crucial for its α-amylase inhibition, since, in comparison with quercetin, which presents a 3-OH group, its α-amylase inhibition significantly decreased (IC_50_ = 138 ± 5 µM). Also, the absence of the 3-Cl atom led to a decrease in the inhibitory activity of flavones, as it is possible to verify through comparing **18a** with luteolin (IC_50_ = 78 ± 3 µM).

The compounds **18a**–**e** also did not inhibit human liver FBPase up to the maximum tested concentration of 200 μM [28], despite their promising activities against α-amylase and α-glucosidase. These results highlight the importance of finding a detailed potential antidiabetic mechanism of action of each flavonoid and, in this way, the possible finding of a specific target-directed therapeutic.

The in vitro inhibitory capacity of the chlorinated flavones **18a**–**e** against DPP-4 was also evaluated [27]. The most effective compounds were **18b** and **18e** (Figure 11), showing similar activities (IC_50_ = 170 ± 10 and 171 ± 8 µM, respectively). Structurally, the presence of an additional 8-Cl substituent in the derivative **18e** does not affect the inhibitory effect of this type of flavones.

### 4.2. 2-(Styryl or 4-Arylbutadienyl)chromones

**Figure 12:**
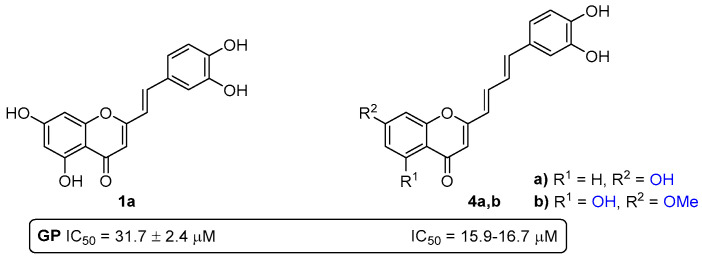
Chemical structures of the most active 2-SC **1a** and 2-[4-(3,4-dihydroxyphenyl)butadienyl]chromones **4a**,**b** as GP inhibitors.

Several chromone derivatives bearing a styryl or a 4-arylbutadienyl group at C-2 were evaluated for their in vitro ability to inhibit GP [32]. The SAR study indicates that the presence and the position of free hydroxy groups at A and B rings is determinant for the inhibitory activity of these two families. Among the 2-SCs, compound **1a**, bearing hydroxy groups at C-5 and C-7 of the A-ring and a catechol moiety on the B-ring (Figure 12), was the most active compound, with an IC_50_ value of 31.7 ± 2.4 µM. Moreover, the 2-(4-arylbutadienyl)chromone **4a** bearing a hydroxy group at C-7 of the A-ring and a catechol moiety on the B ring (Figure 12), and compound **4b** with a hydroxy group at C-5 and a methoxy group at C-7 of the A-ring, and a catechol moiety on the B-ring (Figure 12), were the most active derivatives, with similar IC_50_ values of 16.7 ± 1.5 µM and 15.9 ± 1.1 µM, respectively.

### 4.3. Xanthones

**Figure 13:**
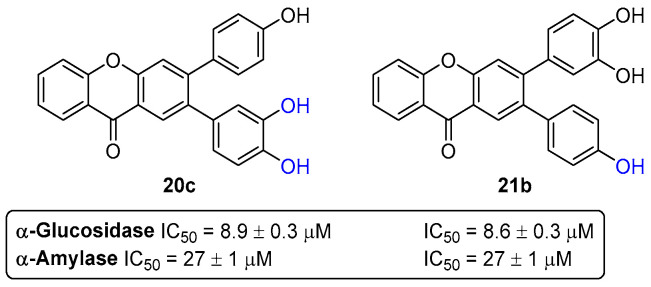
Chemical structures of the lead compounds **20c** and **21b** as dual-target inhibitors of both α-glucosidase and α-amylase.

A series of nine hydroxylated xanthones **19**–**21** (Figure 9) were evaluated as dual-target antidiabetic agents, acting in the inhibition of both α-amylase and α-glucosidase enzymes [33]. The results showed that the xanthones **19**–**21** exhibited a stronger inhibition of α-glucosidase rather than of α-amylase, since all of them (except for the derivative **19a**) present IC_50_ values (8.6–27.4 µM) lower than acarbose, used as a positive control (IC_50_ = 515 ± 19 µM). Particularly, the derivatives **20c** (IC_50_ = 27 ± 1 µM), **21a** (IC_50_ = 23 ± 1 µM), and **21b** (IC_50_ = 27 ± 1 µM), bearing one catechol moiety, were the most active inhibitors of α-amylase, while the xanthones **20c** (IC_50_ = 8.9 ± 0.3 µM), **21b** (IC_50_ = 8.6 ± 0.3 µM), and **21c** (IC_50_ = 9.2 ± 0.4 µM) were the most active against α-glucosidase activity, with IC_50_ values lower than 10 μM. The polyhydroxylated xanthones **20c** and **21b** can be considered as lead dual-target inhibitors of both the α-amylase and α-glucosidase enzymes (Figure 13).

### 4.4. Pyrazoles

**Figure 14:**
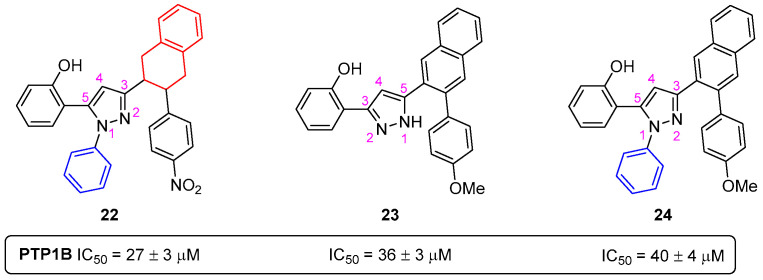
Chemical structures of the most active pyrazoles **22**–**24** as PTP1B inhibitors.

A library of twenty-two pyrazoles was evaluated for the first time as human PTP1B inhibitors [34]. The pyrazole **22**, bearing a phenyl group at N-1, a 2-(4-nitrophenyl)tetralin at C-3 and a phenol at C-5 (Figure 14); pyrazole **23**, holding a phenol at C-3 and a 2-(4-methoxyphenyl)naphthalene at C-5 (Figure 14); and pyrazole **24**, presenting a phenyl group at N-1, a 3-(4-methoxyphenyl)naphthalen-2-yl at C-3 and a phenol at C-5 (Figure 14), were the most active compounds, with IC_50_ values of 27–40 µM, respectively. These findings suggest that the presence of additional benzene rings as functional groups in the pyrazole moiety increases their ability to inhibit PTP1B. The most active compounds showed selectivity over the homologous T-cell protein tyrosine phosphatase (TCPTP).

**Figure 15:**
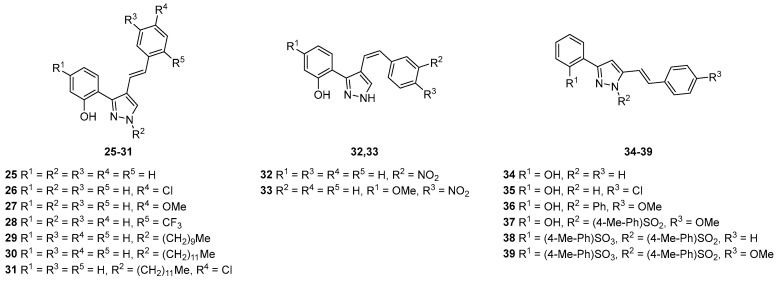
Chemical structures of the tested (*E*)-4-styrylpyrazoles **25**–**31**, (*Z*)-4-styrylpyrazoles **32**,**33**, and 5-styrylpyrazoles **34**–**39** as GP inhibitors.

The inhibitory activity of a group of 4- and 5-styrylpyrazoles **25**–**39** (Figure 15) was evaluated against GP [32]. However, no relevant inhibitory activities were observed up to the highest tested concentration of 50 µM.

## 5. Anticancer Activity

Cancer is one of the main causes of death worldwide, accounting for nearly 10 million deaths in 2020 [35]. Through the decades, the cure rate of patients has increased due to improved early diagnosis and more personalized treatments. Among them, radiation therapy, surgery, immunotherapy, endocrine therapy, gene therapy, and chemotherapy should be highlighted, with the latter being the most widely used either as monotherapy or in combination with other treatments. Despite the overall success of most types of chemotherapies, resistance in most aggressive cancers has increased, which, together with the adverse effects of chemotherapy, has led to the need for the development of new anticancer agents.

**Figure 16:**
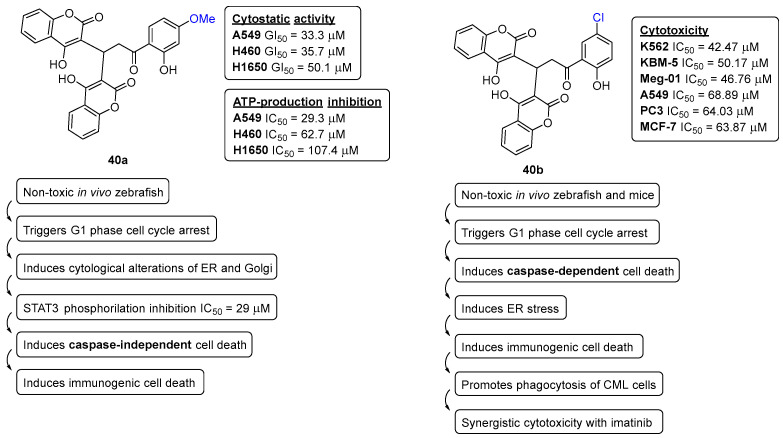
Chemical structures of bis-coumarins **40a**,**b** and their anticancer profile.

One of the main focuses of our research group’s efforts in medicinal chemistry is in the early-stage drug discovery of anticancer small molecules. Two seminal works reported by our research group are focused on synthetic bis-coumarin compounds, more specifically, the **40a**,**b**, and their effects in lung cancer cells with KRAS mutations and myeloid leukemia (CML) cell models (Figure 16).

After a thorough investigation, **40a** was found to strongly inhibit the proliferation of non-small cell lung cancer cells with KRAS mutations through the reduction in aldehyde dehydrogenase expression and abrogated spheroid formation. Subsequent mechanistic investigations showed that **40a** triggers cellular stress, including metabolic catastrophe, mitochondrial stress, and ER/Golgi stress preceded by STAT3 inactivation, inhibiting the STAT3 transactivation and expression of its target genes linked to cell proliferation. It was also demonstrated that **40a** activates sensitization against BH3 mimetics in NSCLC, leading to immunogenic cancer cell death mechanisms. Overall, this compelling evidence supports the potential of bis-coumarin templates as novel candidates for future drug investigation in lung cancer [36].

Driven by the stimulating results of **40a** triggering ER stress in lung cancer, the potential of its chlorinated analogue **40b** (Figure 16) was further investigated against various chronic myeloid leukemia (CML) cell models. Once more, **40b** was proved to trigger ER stress leading to canonical, caspase-dependent apoptosis and the release of danger-associated molecular patterns. The compound **40b** was also capable of inhibiting tumor necrosis factor α-induced activation of nuclear factor-κB, producing synergistic effects upon combination with imatinib, inhibiting the colony formation in vitro and Bcr-Abl+ patient blast xenograft growth in zebrafish [37]. An additional synergistic capacity of **40b** with omacetaxine was observed in imatinib-resistant KBM-5 R cells, resulting in the inhibition of the expression of Mcl-1 and triggering apoptosis [37].

**Figure 17:**
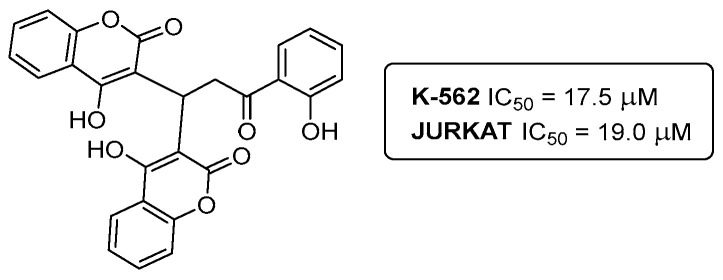
Bis-coumarin **40c** as template for the development of anticancer agents.

Both these seminal publications on the bis-coumarin compounds **40a**,**b** had their starting point many years ago, back into 2014. One of the first studies involving our own synthetic bis-coumarins described their effects on leukemic cell line proliferation and NF-kB regulation [38]. The investigation carried out, in collaboration with Professor Marc Diederich (College of Pharmacy, Seoul National University), started with the synthesis of the template bis-coumarin **40c** (Figure 17). The anti-proliferative effects of compound **40c** on human K-562 (chronic myeloid leukemia) and JURKAT (acute T-cell leukemia) cell lines were assessed, and the results revealed the inhibition of TNFa-induced NF-kB activation in K-562 (IC_50_ = 17.5 μM) and JURKAT (IC_50_ = 19.0 μM) cell lines. Of note, the compound **40c** did not affect the viability of peripheral blood mononuclear cells (PBMCs) from healthy donors, even at concentrations above 100 μM [38]. The bis-coumarin molecule **40c** was deconstructed into fragments as substructures of the bis-coumarin-type compound, 20-hydroxyphenylpropione and 4-hydroxycoumarin, which were completely inactive [38].

**Figure 18:**
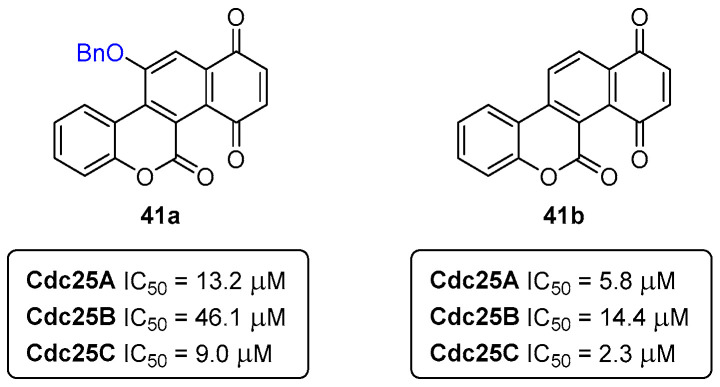
Coumarin derivatives as Cdc25 phosphatase inhibitors.

Cdc25 phosphatases are key enzymes regulating the cell cycle, being a valuable target for cancer treatment. To assess the inhibitory potential of small-molecules towards Cdc25 phosphatases, human glutathione-*S*-transferase (GST)-Cdc25 recombinant enzymes are usually the ideal choice. As demonstrated in previous reports, coumarin derivatives enclose a great potential in the development of alternative cancer therapies. As such, a library of coumarin-based polycycles, like the ones in Figure 18, was screened for their Cdc25 phosphatase-inhibition activity. The screening showed that the coumarins **41a**,**b** (Figure 18) were the most potent phosphatase inhibitors with low micromolar IC_50_ values. More specifically, the benzylated compound **41a** presented IC_50_ (Cdc25A) = 13.2 μM, IC_50_ (Cdc25B) = 46.1 μM, and IC_50_ (Cdc25C) = 9.0 μM, while the derivative **41b** showed IC_50_ (Cdc25A) = 5.8 μM, IC_50_ (Cdc25B) = 14.4 μM, and IC_50_ (Cdc25C) = 2.3 μM [39].

**Figure 19:**
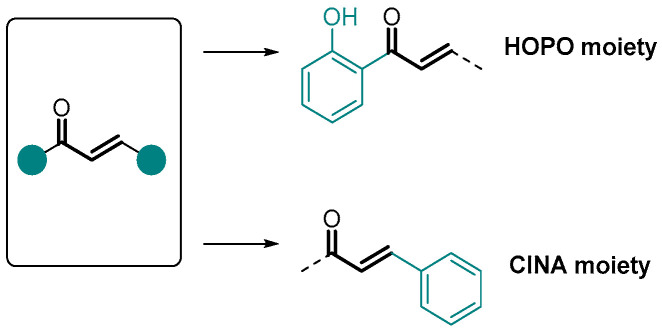
α,β-Unsaturated carbonyl moiety as warhead of covalent inhibitors.

**Figure 20:**
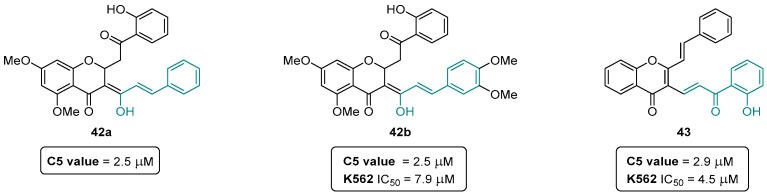
Chromanones **42a**,**b** and 2-SC **43** as potent inducers of the cytoprotective Keap1-Nrf2 signaling pathway.

Covalent inhibitors are being increasingly recognized as an important component in drug discovery and therapeutics. Generally, covalent inhibitors are designed to form a covalent bond with a specific molecular target. The covalent bond can be either reversible or irreversible, depending on the chosen warhead. A number of different warheads have been exploited to target specific amino acid residues, including, among others, cysteine, serine, threonine, tyrosine, and lysine. One of the most commonly used warheads is the α,β-unsaturated carbonyl moiety (Figure 19), which can be found in several covalent inhibitors in clinical trials [40,41,42]. Some variations in these Michael acceptor moieties have also been reported, such as the (2-hydroxyphenyl)-3-oxoprop-1-enyl (HOPO) and the cinnamoyl (CINA) (Figure 19). In this sense, the chromanones **42a**,**b**, as well as the 2-SC **43** were specifically designed to have the HOPO and CINA pharmacophoric moieties (Figure 20).

The chromanones **42a**,**b** revealed a C5 value of 2.5 μM [C5 value, concentration causing a five-fold induction of luciferase activity (= Nrf2 activity)], being more potent than the positive controls xanthohumol (XN) C5 = 7.8 μM and sulforaphane (SFN) C5 = 4.8 μM [43]. The 2-SC **43** showed a slightly higher C5 value of 2.9 μM [43]. In an additional assay, the chromanone **42b** and 2-SC **43** were also shown to be the more potent compounds inhibiting leukemia K562 cell proliferation, with IC_50_ values of 7.9 ± 2.6 μM and 4.5 ± 1.9 μM, respectively, with no appreciable effect on normal peripheral blood mononuclear cells [43]. Despite the encouraging results of the compounds **42a**,**b** and **43**, it still unknown if the mechanism of action is indeed covalent or non-covalent, and, therefore, further mechanistic studies are still required.

**Figure 21:**
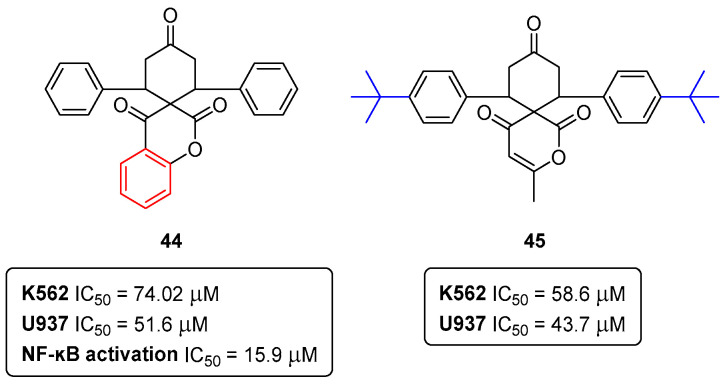
Spiro(lactone-cyclohexanones) as antiproliferative agents in leukemia cell lines.

It is commonly accepted that the small-molecule inhibition of nuclear factor-ĸB (NF-ĸB) is quite an interesting strategy to improve cancer chemotherapy. In this field of research, our group reported collaborative work in the anti-proliferative, cytotoxic, and NF-ĸB inhibitory properties of spiro(lactone-cyclohexanone) synthetic compounds in human leukemia [44]. The spiro(lactone-cyclohexanone) derivatives **44** and **45** (Figure 21) were revealed as the most effective in the inhibition of the proliferation of the human leukemia cell lines K562 and U937, with IC_50_ values of 74.02 ± 4.10 μM (K562) and 51.6 ± 4.2 μM (U937) for the derivative **44**, and 58.6 ± 4.2 μM (K562) and 43.7 ± 1.5 μM (U937) for compound **45** [44]. Additionally, the spiro(lactone-cyclohexanone) **44** was also capable of reducing TNFα-stimulated NF-ĸB activation, with an IC_50_ of 15.9 ± 4.0 μM [44].

**Figure 22:**
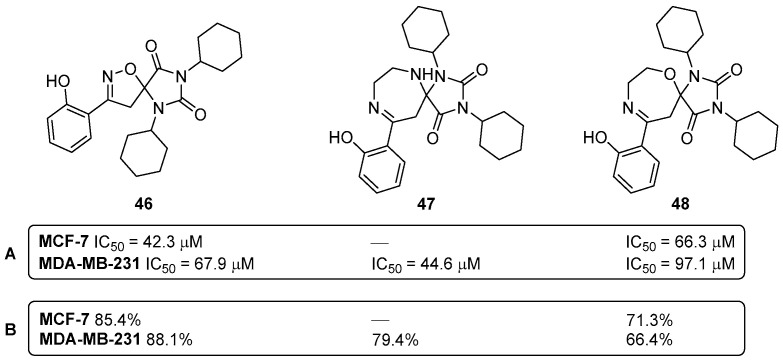
Effective spiro-*bis*heterocyclic compounds **46**–**48** against proliferation (**A**) and apoptosis (**B**) of human breast cancer cell lines.

Within the spirocyclic chemotype compounds, the spiro-heterocyclic surrogates have also been showing promising results in the chemotherapy of various cancer types. Back in 2016, a series of oxygen and nitrogen spiro-*bis*heterocycles were studied for their effects on the in vitro proliferation and apoptosis of human breast cancer cell lines (MCF-7 and MDA-MB-231) [45]. From the screened library, the three compounds **46**–**48** stood out as the most effective ones (Figure 22).

Compounds **46** and **48** showed a dose-dependent decreasing effect in cell proliferation and induced apoptosis in the MCF-7 and MDA-MB-231 cell lines, while the derivative **47** was only active towards MDA-MB-231 [45]. In this study, it was also reported that the compounds **46**–**48** cause apoptosis by targeting p53–MDM2 interaction. It was also investigated and concluded that the spiro-heterocyclic compounds **46**–**48** promote apoptosis via the p53-independent pathway(s) [45].

**Figure 23:**
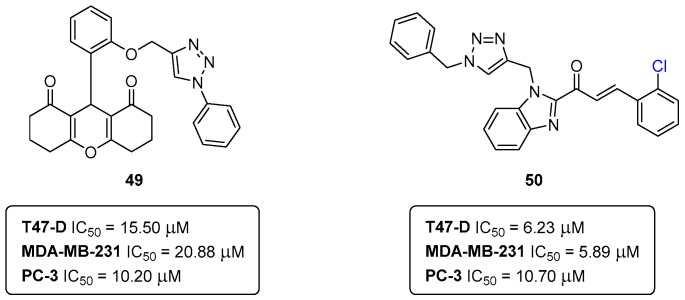
1,2,3-Triazole-xanthenediones and triazole-benzimidazole-chalcones as anticancer agents.

More recently, other collaborative projects have led to the expansion of the chemical space of anticancer agents, namely, towards 1,2,3-triazole-xanthenediones and triazole-benzimidazole-chalcones (Figure 23) [46,47]. The reported library of 1,2,3-triazole-xanthenediones was screened in breast cancer (T47-D and MDA-MB-231) and prostate cancer (PC3) cell lines. The compound **49** (Figure 23) was the most generally effective compound, with IC_50_ (T47-D) = 15.50 ± 1.59 μM, IC_50_ (MDA-MB-231) = 20.88 ± 0.20 μM, and IC_50_ (PC3) = 10.20 ± 0.22 μM [46].

The library of triazole-benzimidazole-chalcones was also screened in the same types of cancer cells. Interestingly, the chlorinated derivative **50** (Figure 23) was the best-performing compound with IC_50_ (T47-D) = 6.23 ± 1,03 μM, IC_50_ (MDA-MB-231) = 5.89 ± 1.35 μM and IC_50_ (PC3) = 10.70 ± 1.25 μM [47].

The common structural motif among the compounds **49** and **50** is the 1,2,3-triazole. Actually, the 1,2,3-triazole ring is a major pharmacophore system among nitrogen-containing heterocycles, displaying relevant roles either as key pharmacophores or linkers [48].

**Figure 24:**
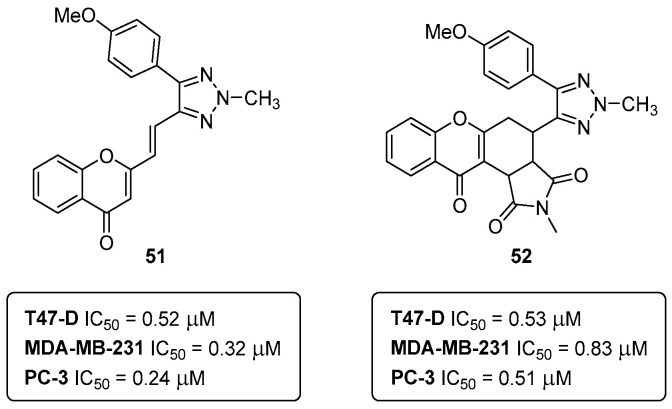
Chromone-1,2,3-triazole hybrid compounds targeting breast and prostate cancer cells.

In a recently published study (2023), eight in-house chromone-based compounds were screened for their effects in breast (T-47D and MDA-MB-231) and prostate (PC3) cancer cell lines, and in non-cancerous human mammary epithelial cells (HuMECs). Interestingly, the compounds with better performance in cancer cells were those with the 1,2,3-triazole moiety, **51** and **52** (Figure 24) [49].

Both compounds presented very good potencies in three of the tested cell lines, i.e., **51**, IC_50_ (T-47D) = 0.52 μM, IC_50_ (MDA-MB-231) = 0.32 μM, and IC_50_ (PC3) = 0.24 μM; **52**, IC_50_ (T-47D) = 0.53 μM; IC_50_ (MDA-MB-231) = 0.83 μM and IC_50_ (PC3) = 0.51 μM [49]. In breast (MDA-MB-231) and prostate (PC3) cell lines, the compounds **51** and **52** were actually more potent than the positive control (doxorubicin IC_50_ 1.51 μM for MDA-MB-231 and IC_50_ = 0.73 μM for PC3). Notably, the IC_50_ of the compounds **51** and **52** was 24–388 times lower in non-cancerous HuMECs when compared to the IC_50_ of doxorubicin (0.57 μM) [49]. The compounds **51** and **52** have different mechanisms of action; while the chromone-1,2,3-triazole **51** induces G2/M arrest in T-47D, MDA-MB-231, and PC3 cells, compound **52** had no effect on the cell cycle [49]. In terms of the structure–activity relationships (SARs), the main conclusion is that the installation of a tetrahydroisoindole-1,3-dione moiety (Figure 24) is irrelevant for potency in all the tested cancer cell lines, but it is important in lowering the cytotoxic effect in HuMECs (IC_50_ = 221.35 μM) [49]. Of note, the hybrid compounds **51** and **52** are the most potent anticancer agents in the more than twenty years of medicinal chemistry research in our lab, and, therefore, the most promising drug candidates.

**Figure 25:**
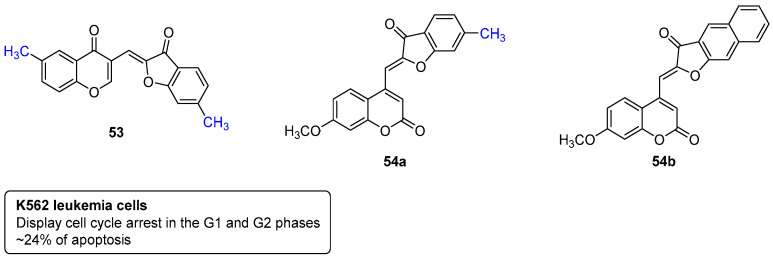
Chromone– and coumarin–aurone hybrids targeting K562 human leukemia cells.

Years ago, chromones were also combined with additional frameworks such as aurones (2-benzylidene-benzofuran-3(*2H*)-ones) (Figure 25). In the same project, the aurones were further combined with coumarins (Figure 25), and both libraries of compounds were screened for their anti-cancer activities in K562 human leukemia cells [50]. Most of the screened compounds were capable of blocking the K562 cell cycle in the G1, S, or G2 phase, with the compounds **53** and **54a**,**b** also being capable of inducing a high apoptosis rate (~24%) [50].

**Figure 26:**
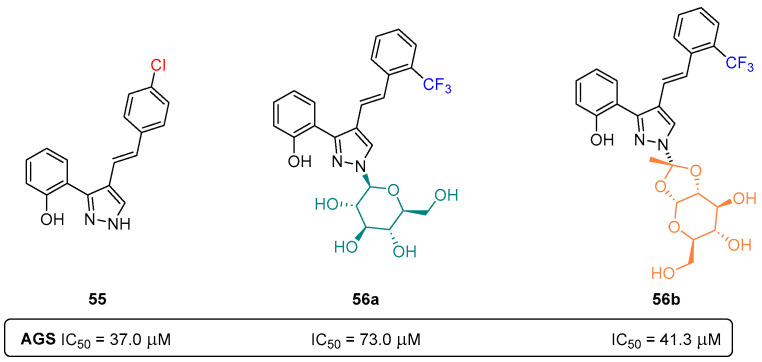
Glycosylated- and non-glycosylated pyrazoles as anticancer agents.

Another important class of azoles are the pyrazoles, five-membered heterocyclic aromatic rings with three carbons and two adjacent nitrogen atoms. Charting the long history of our research group in the chemistry of pyrazoles, in 2019, a series of styrylpyrazole-glucosides (Figure 26) was reported [51]. This new class of compounds, as well as their non-glycosylated precursors, were further evaluated for their cytotoxic potential against human gastric adenocarcinoma AGS cells and healthy MRC-5 lung fibroblasts.

The preliminary cytotoxic evaluation human gastric adenocarcinoma AGS cells revealed the non-glycosylated pyrazole **55** (Figure 26) as the most potent compound among the tested analogues (IC_50_ = 37.0 μM), while the glycosylated compounds **56a**,**b** were less potent (**56a** IC_50_ = 73.0 μM; **56b** IC_50_ = 41.4 μM). This suggests the more significant role of Cl-substitution over either the CF_3_-substitution or the glycosyl moiety (Figure 26). As for the non-cancer cell line (MRC-5 lung fibroblasts), the most potent pyrazole **55** also displays a certain degree of toxicity (IC_50_ = 45.0 μM), being only slightly more active towards the tumor cell line, AGS [51].

**Figure 27:**
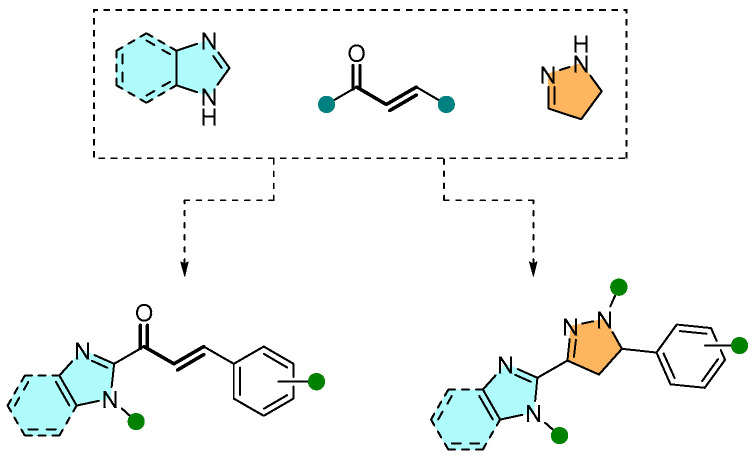
Design strategy of chalcone-type and 2-pyrazoline compounds incorporating an (benz)imidazole moiety.

Within the large family of azoles, the 2-pyrazolines and (benz)imidazoles also show important biological relevance. In a recent collaborative effort, our research group reported the design of chalcone-type and 2-pyrazoline derivatives bearing an (benz)imidazole moiety (Figure 27), as well as their effects on human lung (A549) and stomach (AGS) cancer cell lines, being additionally evaluated in the non-cancer human lung fibroblast (MRC-5) cell line [52].

**Figure 28:**
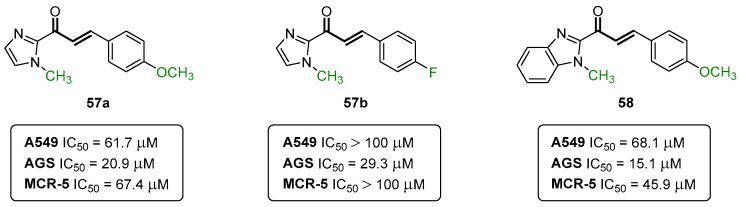
Chalcone-type compounds identified as promising anticancer agents toward the AGS cell line.

An appreciable library of fifteen compounds covering several substitution patterns were synthesized and screened. While 2-pyrazoline derivatives were devoid of toxicity in all cell lines used, the chalcones bearing the (benz)imidazole ring in the β position of the carbonyl group **57a**,**b** and **58** (IC_50_ = 15.1–29.3 μM) were found to be toxic toward the AGS cell line (Figure 28), while only compounds **57a** and **58** were toxic for A549 (IC_50_ = 61.7–68.1 μM). Importantly, these compounds were considerably less toxic toward non-cancer cells. Further mechanistic investigations on compounds **57a**,**b** and **58** revealed the trigger loss of cell viability and mitochondrial membrane potential, while eliciting morphological traits compatible with refractory cancer disease (RCD). All three compounds were less toxic when incubated in the presence of a pan-caspase inhibitor and compounds **57b** and **58** were shown to increase the activity of caspase-3 [52].

**Figure 29:**
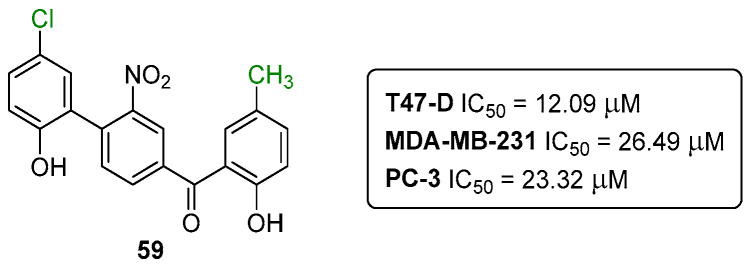
Structure of aryl-benzophenone **59**.

Another important class of anti-cancer agents are the benzophenones. Several examples of both natural and synthetic analogues can be found in the literature. In 2019, our research group reported additional examples of anticancer aryl-benzophenones targeting breast and prostate cancer cell lines [53]. The most prominent compound was the benzophenone **59** (Figure 29), showing IC_50_ values of 26.49 ± 0.95, 12.09 ± 1.07, and 23.32 ± 0.05 μM, for the MDA-MB-231, T47-D, and PC-3 cell lines, respectively [53].

Of note, the benzophenone **59** showed induced cell cycle retardation only in prostate cancer cells, suggesting cell type-dependent effects that may be related to the compound acting on molecular targets which are differentially expressed in breast and prostate cancer cells. Although, more detailed molecular studies are required to provide clarifications on the use of benzophenone **59** as a pharmacophore and to understand its specific action on mitochondrial membrane, protein, and glycoprotein interactions [53].

**Figure 30:**
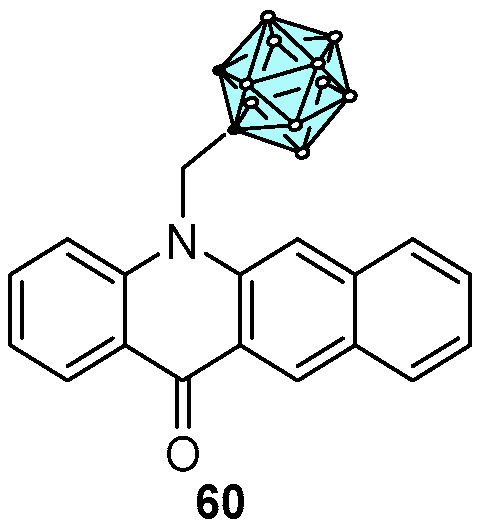
Benzo[*b*]acridin-12(7*H*)-one **60** as lead structure for BNCT.

In 2014, one of the most interesting research projects from our group in medicinal chemistry targeting cancer was published. In this work, the synthesis of benzo[*b*]acridin-12(7*H*)-ones with tethered carboranyl moieties was described (Figure 30), as well as their potential as boron neutron capture therapy (BNCT) agents in cancer treatment [54].

Remarkably, compound **60** (Figure 30) showed no cytotoxicity in U87 human glioma cells, a clinically relevant tumor cell line for BNCT, in concentrations ranging 5–200 μM. This is an important parameter in BNCT so that boron concentrations within tumors can be maximized. The subsequent evaluation of benzo[*b*]acridin-12(7*H*)-one **60** as a new BNCT agent demonstrated that it enters the cells and deposits an adequate amount of B atoms (2.8 × 10^10 10^B atoms per cell) superior to the recommended concentration of 10^8^–10^9 10^B atoms, demonstrating considerably high activity in the U87 cells upon neutron irradiation [54].

## 6. Anti-Alzheimer Activity

Alzheimer’s disease (AD) is the most prevalent neurodegenerative disorder, quickly becoming a major healthcare and economic problem to deal with in modern societies. As for the majority of neurodegenerative diseases, AD is age-related and mainly affects people over 65 years old. In 2022, an estimated 32 million people worldwide suffered with AD, and this figure is expected to rise to 131.5 million in 2050 [55].

The multifactorial nature of AD has led to the discovery of several targets or target combinations in order to find effective treatments. Among many theories behind the disease pointing to glutamate excitotoxicity [56], oxidative stress [57], and biometal dyshomeostasis [58] as causative triggers of the underlying neurodegeneration (reviewed elsewhere [59]), the amyloid-β (Aβ) plaques and tau fibrils are hypothesized as the major contributing factors [60,61]. The mainstream concept though is clearly the amyloid cascade hypothesis (formulated by Hardy and Higgins), which explains that the aggregation of Aβ species causes neural death and eventually progression to AD [62,63].

**Figure 31:**
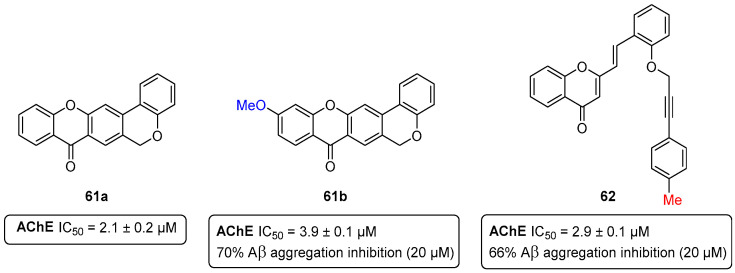
Single- and dual-target lead compounds towards AChE and Aβ aggregation.

The “cholinergic hypothesis” [64], which was the first and most studied approach describing AD pathophysiology, is based on compelling evidence mainly regarding three aspects: (i) the nucleus basalis of Meynert in the basal forebrain undergoes severe neurodegeneration in AD; (ii) the presynaptic cholinergic markers in the cerebral cortex are depleted seriously in AD; and (iii) cholinergic antagonists impair memory, whereas agonists alleviate cognitive deficits [65]. The theory behind the “cholinergic hypothesis” led to the development of not only several acetylcholinesterase inhibitors (AChEIs), but also many examples of dual- or multi-target compounds combining inhibitory effects in AChE with other AD-related targets (the so-called multi-target cholinesterase inhibitors) [65,66]. One of the most common strategies to develop multi-target directed ligands (MTDLs) envisioning anti-AD agents, is based on the design of AChE and Aβ inhibitors [66,67]. On the basis of this, in 2021, we described first-in-class AChE and Aβ aggregation dual-inhibitors based on the chromeno[3,4-*b*]xanthone scaffold (Figure 31) [68]. Also, the (*E*)-2-[2-(propargyloxy)styryl]chromone precursors were screened, and, from this study, three lead compounds were identified (Figure 31).

The non-substituted chromeno[3,4-*b*]xanthone **61a** emerged as a lead single-target AChEI, with an IC_50_ value of 2.1 μM (Figure 31). Upon the installation of a methoxy group (compound **61b**), the AChE inhibition slightly decreased to IC_50_ = 3.9 μM, while the anti-aggregation activity increased up to 70% inhibition at 20 μM (Figure 31). Concerning the (*E*)-2-[2-(propargyloxy)styryl]chromone precursors, their overall activity was limited, with the exception of compound **62**, which presented an IC_50_ (AChE) = 2.9 μM and 66% of Aβ aggregation inhibition at 20 μM (Figure 31) [68].

**Figure 32:**
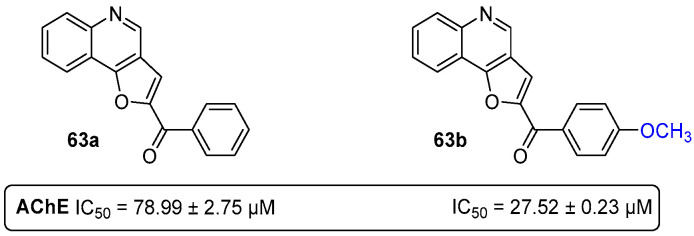
2-Aroylfuro[3,2-*c*]quinolines as AChE inhibitors.

In a different research project, 2-aroylfuro[3,2-*c*]quinolines (Figure 32) showed reasonable potential as AChE inhibitors, although much less potent than the above mentioned chromeno[3,4-*b*]xanthones [69]. The non-substituted 2-aroylfuro[3,2-*c*]quinoline **63a** showed an IC_50_ = 78.99 μM, while its methoxylated analogue **63b** was 2.9-fold more potent (IC_50_ = 27.52 μM) as an AChEI (Figure 32) [69].

**Figure 33:**
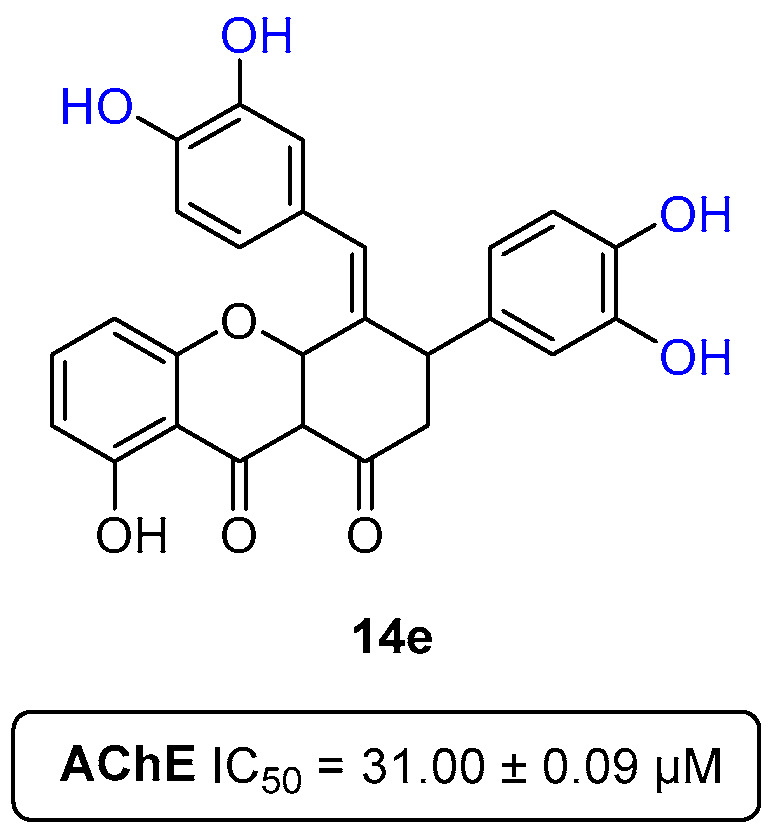
Xanthenedione with two catechol moieties as AChE inhibitor.

Back in 2014, our group reported a different type of compound with decent potency against AChE, the xanthenediones. Within a small library of six tested compounds (Figure 5), xanthenedione **14e** (Figure 33), having two catechol moieties, showed an IC_50_ value of 31.0 ± 0.09 μM [15].

When it comes to AD-targeting compounds, steroids are perhaps the last class of compounds that comes to mind. It happens that steroids enclose a great unexplored potential to develop alternative therapies for AD, in two ways: (i) they have protein aggregation inhibition capacity, Aβ above all [70]; and (ii) they can be used as an anchor to the neuronal cell membrane−lipid rafts enhancing the blood–brain barrier (BBB) permeability [71].

**Figure 34:**
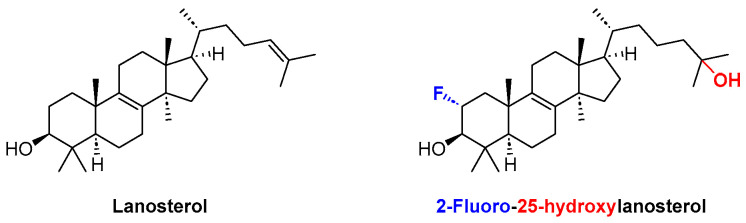
Structures of lanosterol and 2-fluoro-25-hydroxylanosterol.

It all started in 2015, when lanosterol (Figure 34) was reported as an efficient inhibitor of crystallin aggregation in vivo, due to its amphiphilic nature which enables it to intercalate into and coat hydrophobic core areas of large protein aggregates, allowing these aggregations to gradually become water-soluble again [72,73]. The efficacy of lanosterol against crystallin aggregation was further enhanced through the chemical installation of a fluorine and a hydroxy group in positions C-2 and C-25 of the basic lanosterol scaffold, respectively (Figure 34) [74].

The potential of lanosterol as an inhibitor of general protein aggregation was demonstrated later against the Aβ peptide aggregation [70]. Molecular dynamics simulations with the amyloidogenic segment core (KLVFFA) of Aβ showed two types of inhibition mechanisms: (i) lanosterol entangles with peptides to establish a hydrophobic moiety with residues Phe-19 and Phe-20; and (ii) it can also interfere with the steric zipper interaction at the β-sheet−β-sheet interface [70]. Nevertheless, the thioflavin (ThT) fluorescence assay and AFM imaging showed that the minimum lanosterol concentration necessary to effectively inhibit the Aβ peptide self-aggregation in vitro must be 200 μM (Aβ/lanosterol ratio = 1:8), which is a relatively high value for potential use as a drug [70].

**Figure 35:**
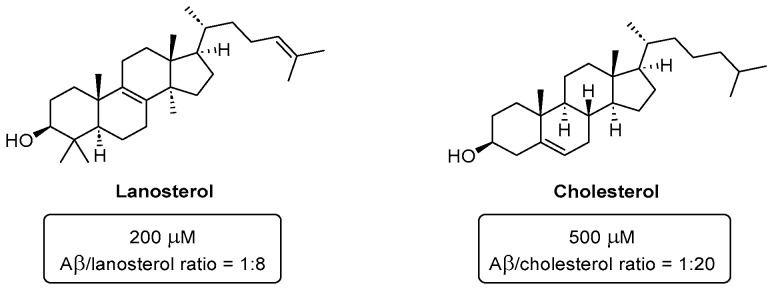
Structures of lanosterol and cholesterol and their Aβ/steroid ratios for aggregation inhibition.

The most curious conclusion of all these studies is that cholesterol (Figure 35) was always a much weaker inhibitor of either crystallin or Aβ aggregation (in the latter case, 500 μM are necessary to achieve reasonable inhibition) [70,72]. Despite the structural similarities between lanosterol and cholesterol (both are amphiphilic compounds), the later one is less hydrophobic and therefore less effective against protein aggregation. Thus, it is conceivable that, if the structure of cholesterol was appropriately modified, we might achieve even better effectiveness than lanosterol towards the inhibition of Aβ aggregation.

**Figure 36:**
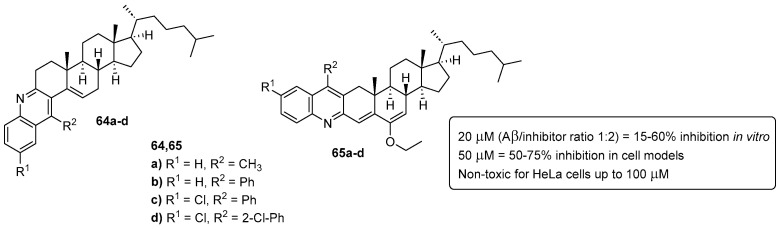
Quinoline–cholesterol hybrids as effective inhibitors of Aβ and random protein aggregation.

Having that in mind, through a “framework combination” design strategy, we prepared two types of quinoline–cholesterol hybrid compounds **64** and **65** (Figure 36) which demonstrated high capacity in the inhibition and reversion of Aβ aggregation in vitro and in cell models [75]. At 20 μM (Aβ/inhibitor concentration ratio = 1:2), compounds **64** and **65** showed a 15–60% inhibition range of ThT-monitored Aβ1-42 in vitro self-aggregation [75].

In cell models, namely, SH-SY5Y neuronal-like cells incubated with pre-aggregated Aβ1-42 synthetic peptide and HeLa cells expressing the protein aggregation sensor HSP27:GFP [76] with nilotinib (NTB) as a proteostasis impairment inducer, the hybrid compounds **64** and **65** have demonstrated a disaggregation capacity of ∼50–75% [75].

## 7. Bioimaging

**Figure 37:**
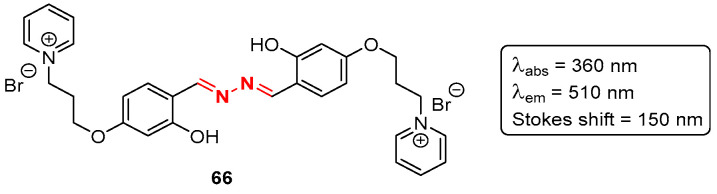
Fluorescent light-up probe **66** for detection of protein aggregates.

Bioimaging is generically defined as a noninvasive process of visualizing biological activity in a specific period of time. Over the years, several imaging techniques, such as positron emission tomography (PET), magnetic resonance imaging (MRI), and single photon emission computed tomography (SPECT), have been developed both for clinical applications and fundamental research. In particular, fluorescence-based techniques have been useful to study several types of protein aggregates [77,78,79], and developing improved fluorophores for protein aggregation is of the utmost importance, for example, in the aging population. In this context, colleagues in our research group developed an azine-based probe **66**, decorated with pyridine cationic groups which assure its solubility in water and biological media (Figure 37) [80].

The probe **66** presented an absorption and emission wavelength of 360 and 510 nm, respectively, and a very large Stokes shift (150 nm), with almost no overlap between the absorption and emission bands. Additionally, the azine-based probe **66** showed an enhancement of its emission intensity upon interaction with DNA as well as upon complexation with cyclodextrins. Moreover, co-staining experiments with Proteostat^®^ in HeLa cells revealed 54% of the co-localized population with probe **66** [80].

**Figure 38:**
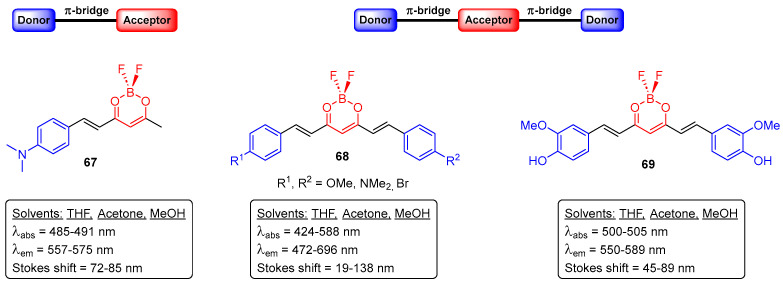
Curcumin-based molecular probes for staining *Fusarium oxysporum*.

Fungi are eukaryotic organisms that morphologically can be assigned to two main groups: unicellular fungi (yeasts) and filamentous fungi (molds). The typical fungal cell has a rigid cell wall containing chitin, glucans, or chitosan, which serves as a protective layer. As consequence, it is quite difficult for imaging probes to cross this wall and, therefore, the fluorescence imaging of fungi is hard, and the synthetic organic dyes available to do so are limited [81]. Very recently, our research group developed additional curcumin-based molecular probes **67**–**69**, for the staining of a filamentous fungus (*Fusarium oxysporum*) (Figure 38), based either in the donor–acceptor (D–A) or donor–acceptor–donor (D–A–D) architectures [82].

The native fluorescent properties of the represented compounds **67**–**69**, i.e., absorption (λ_abs_) and emission (λ_em_) wavelengths ranged between 424 and 588 nm and 472 and 696 nm, respectively, with a very small shift between the different solvents [82]. The reported probes were found to be non-toxic and capable of entering the fungi cells. Also, they selectively accumulate in sub-cellular compartments, and their visualization is enabled through using different filter sets, depending on the dye used [82].

**Figure 39:**
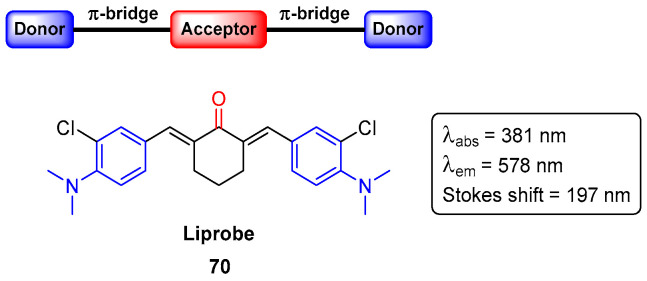
Chemical structure of liprobe with D–A–D.

It is commonly known that lipids are an important class of biomolecules, and are extremely important in metabolic and signaling processes. Despite their biological relevance, the abnormal accumulation in cells and tissues can lead to the development of the so-called lipid-based disorders (or lipidosis) [83]. As such, the early detection of these lipid aggregates is so important for diagnosis and drug screening purposes. One of the best ways to detect lipid aggregates is using fluorescent probes, which are not widely available at a low cost nor are they easy to use. Relying on the group’s experience in the design and synthesis of fluorophores, the so-called liprobe **70** (Figure 39) [84], with a D–A–D architecture, was reported in 2022 [85].

The cyclic-core fluorophore liprobe **70** absorbs at 381 nm and emits at 578 nm, with a quantum yield of 1% in dichloromethane. Liprobe **70** was shown to be physiologically inert in cells, not toxic to living zebrafish embryos, and differentially stained the muscle and bone tissues. Furthermore, confocal and cell-based high content screens revealed that liprobe **70** is able to selectively detect lipid droplets and ceramide loads in normal and Farber’s disease human fibroblasts, respectively. All this evidence demonstrates the potential of liprobe **70** for the establishment of a gold standard to be used in high content screening assays for the preliminary diagnosis of Farber’s disease and, potentially, of other lipidoses [85].

## 8. Other Biological Activities

Infectious diseases have had a profound impact not only on human health but also on the course of history. Despite the continuous discovery and development of anti-infective agents, dating back to the discovery of penicillin, infectious diseases of many kinds still chase even the most developed societies. In this century, the World Health Organization (WHO) declared fourteen infectious disease-related public health emergencies, including SARS, Influenza H1N1, Ebola, MRSA, MERS, Zika, SARS-CoV-2, and monkeypox. On the other hand, these health emergency declarations do not include the chronic infectious diseases, such as HIV, HCV, HBV, HSV, and not to mention bacterial infections or even neglected infectious diseases.

**Figure 40:**
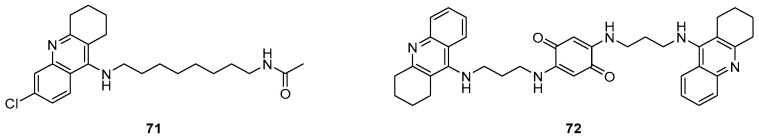
VS hit templates based on the tetrahydroacridine scaffold.

Leishmaniosis is a major health problem, mainly affecting people from developing countries from the tropics, subtropics, and the Mediterranean basin, with an estimated world prevalence of 12 million cases [86]. Recently, our research group extended its medicinal chemistry efforts to the development of antileishmanial agents based on the 1,2,3,4-tetrahydroacridine scaffold [87]. The approach started with a virtual screening (VS) campaign of natural compounds (99 K) against a specific enzyme of the parasite, the *S*-adenosylmethionine decarboxylase (AdoMet DC). A large number of hit compounds were obtained, and upon the retrosynthetic analysis of their complexity, two tetrahydroacridine templates **71** and **72** were selected for the further preparation of analogues (Figure 40) [87].

**Figure 41:**
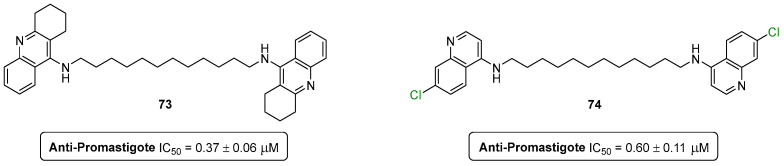
Antileishmanial lead compounds based on tetrahydroacridine and quinoline scaffolds.

A library of forty compounds was screened for its antileishmanial activity against *L. infantum* promastigotes. The screened compounds showed activities in the low micromolar range (IC_50_ = 0.37–14.17 μM). Compounds **73** (IC_50_ = 0.37 ± 0.06 μM) and **74** (IC_50_ = 0.60 ± 0.11 μM) emerged as the most potent analogues against the parasite (Figure 41). However, compound **73** showed a high level of cytotoxicity (99.7% at 10 μM), while the quinoline analogue **74** was much less toxic (EC_50_ = 11.69 ± 3.96 μM), presenting a therapeutic index (TI) of 19.48. This means that the replacement of the tetrahydroacridine for a chloro-quinoline surrogate resulted in much lower toxicity levels, not compromising the antileishmanial activity [87].

Bacterial infections are recognized by WHO as one of the top 10 global public health threats due to ever-increasing anti-microbial resistance. According to a recent report on antimicrobial resistance, about 0.7 million deaths are caused by AMR annually, and this number is expected to increase to 10 million by 2050 [88,89]. Therefore, the discovery of new antibacterial agents is urgent to combat increasing bacterial resistance.

**Figure 42:**
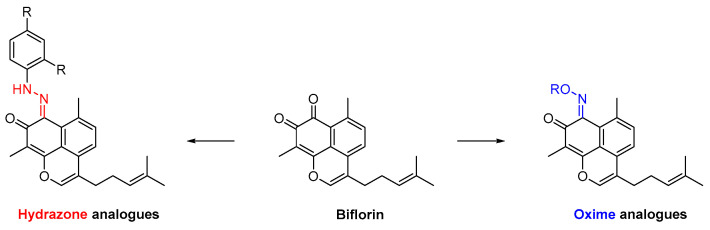
Hydrazone and oxime analogues of biflorin.

In this context, a series of nitrogenated derivatives of biflorin (Figure 42) were synthesized through a reaction with hydrazines and hydroxylamines, and screened for their antibacterial activity against six Gram-positive and Gram-negative bacterial strains [90].

**Figure 43:**
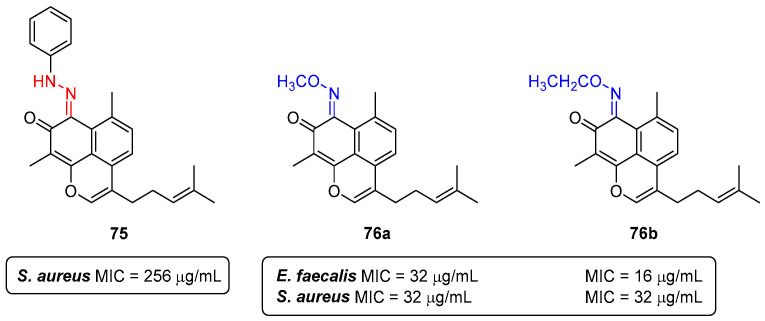
Best performing antibacterial compounds, hydrazone **75** and oximes **76a**,**b**.

All the screened nitrogen analogues demonstrated satisfactory minimum inhibitory concentration (MIC) values. The most promising compounds were the hydrazone **75**, with an MIC value of 256 μg/mL for *Staphylococcus aureus*; and oximes **76a**,**b**, with MIC values of 32 μg/mL and 16 μg/mL for *Enterococcus faecalis*, respectively; and *Staphylococcus aureus*, with MIC values 32 μg/mL, both (Figure 43) [90].

**Figure 44:**
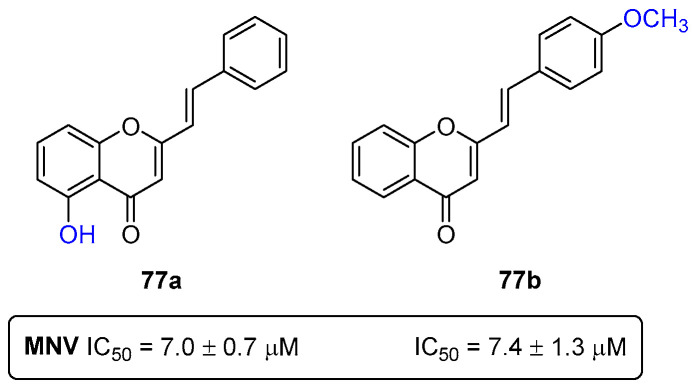
Most effective anti-norovirus (*E*)-2-SC **77a**,**b**. MNV = murine norovirus.

Among several viral infections known by mankind, the human noroviruses (NoV) are recognized as the most frequent cause of outbreaks and sporadic cases of acute gastroenteritis. Despite some developments in vaccine development clinical trials, there is still no available vaccine against noroviruses [91]. Therefore, the discovery of anti-norovirus drugs is an unmet medical need. In this field, back in 2010, our research group reported a series of (*E*)-2-SC with promising anti-norovirus activity [92]. From the twelve screened compounds, the (*E*)-2-SCs **77a**,**b** (Figure 44) were the most potent antiviral agents, with IC_50_ values of 7.0 ± 0.7 μM and 7.4 ± 1.3 μM, respectively [92].

The presence of two specific substituents (the OH and OCH_3_) was crucial for potent antiviral activity. A first insight into the mechanism of action of both (*E*)-2-SCs was proposed. The addition of the compounds at different timepoints revealed that compounds **77a**,**b** interfere more with the steps of the viral life cycle that follow the entrance of the virus in cells [92].

## 9. Conclusions

In this review, we took a survey on more than twenty years of research in medicinal chemistry, developed in Artur Silva’s research group. Several types of compounds were included, such as flavonoids, chalcones, chromones, coumarins, xanthones, quinolines, acridine and acridones, spirocyclic compounds, chromeno[3,4-*b*]xanthones, steroids, and curcumin analogues. All these chemically diverse scaffolds displayed promising biological activities ranging from the traditional antioxidant, anti-inflammatory, and anticancer to the more recent antidiabetic, anti-Alzheimer, and anti-leishmaniosis, passing by the development of imaging tools.

From our point of view, three lead compounds developed by our research group de-serve individual highlights.

The first is the flavonol **10e** (having two catechol moieties at the A- and B-rings), which is the most advanced in-house compound in the antidiabetic drug discovery pipeline. Actually, flavonol **10e** was initially designed as an antioxidant, but repurposed later as an antidiabetic lead, due to its high inhibition of α-glucosidase in vitro as well as in a Caco-2/TC7 cellular model. In 2021, this compound entered preclinical animal model studies.

The second well-deserved highlight goes to the chromone-triazole compound **51**, which was the most potent anticancer compound ever produced in our laboratory. Compound **51** showed IC_50_ values ranging from 0.24 to 0.52 µM, for breast (T-47D and MDA-MB-231) and prostate (PC3) cancer cell lines, being more potent than doxorubicin in the MDA-MB-231 and PC3 cell lines.

The third and final highlight should be given to liprobe **70**, which is the first imaging tool ever patented by our research group. Liprobe **70** holds a European Patent (WO/2017/182945) and shows potential to be a gold standard to be used in the preliminary diagnosis of Farber’s disease and, potentially, of other lipidoses.

## Data Availability

Data sharing is not applicable.
